# Stability of sample extracts of vitamin D_3_ metabolites after chemical derivatization for LC–MS/MS analysis

**DOI:** 10.1007/s00216-022-04409-5

**Published:** 2022-11-07

**Authors:** Anastasia Alexandridou, Dietrich A. Volmer

**Affiliations:** Bioanalytical Chemistry, Department of Chemistry, Humboldt University Berlin, Brook-Taylor-Str. 2, 12489 Berlin, Germany

**Keywords:** Vitamin D_3_ metabolites, 25-Hydroxyvitamin D_3_, LC–MS/MS, Stability, Chemical derivatization

## Abstract

Liquid chromatography/tandem mass spectrometry (LC–MS/MS) is widely used to determine vitamin D_3_ metabolites in biological samples. The ionization efficiencies of these metabolites, however, are poor under electrospray ionization conditions. Moreover, the chromatographic separation of multiple vitamin D metabolites and their epimers can be challenging. For these reasons, chemical derivatization reagents are often used to improve sensitivity and selectivity of analysis. While the derivatization schemes have been proven to be very effective, one missing aspect is the investigation of the stability of the chemical derivatization products in stored sample extracts. In this study, we investigated the long-term stability of several vitamin D_3_ metabolites after 1 and 3 months of storage at − 20 °C. Five vitamin D_3_ metabolites were examined after derivatization with seven different derivatization reagents. Generally, Amplifex products were the most stable in the long term in our study with 11–20% degraded after 1 month of storage and 14–35% after 3 months. The stabilities for some of the metabolites′ 4-[2-(6,7-dimethoxy-4-methyl-3-oxo-3,4-dihydroquinoxalyl)ethyl]-1,2,4-triazoline-3,5-dione (DMEQ-TAD), 2-fluoro-1-methylpyridinium *p*-toluenesulfonate (FMP-TS), isonicotinoyl chloride (INC) and 4-phenyl-1,2,4-triazoline-3,5-dione acetylated (PTAD-Ac) products were also acceptable after 1 month of storage. Other derivatized metabolites, however, degraded extensively already after 1 month of storage, such as 4-phenyl-1,2,4-triazoline-3,5-dione (PTAD) (54–72% degradation) and 2-nitrosopyridine (PyrNO) (32–100% degradation). Importantly, for every metabolite, there was an optimum derivatization reagent that met the criteria of stability proposed by international regulatory bodies after 1 month of storage. Some derivatives were stable for even up to 3 months of storage, with degradation of less than 15%.

**Graphical abstract:**

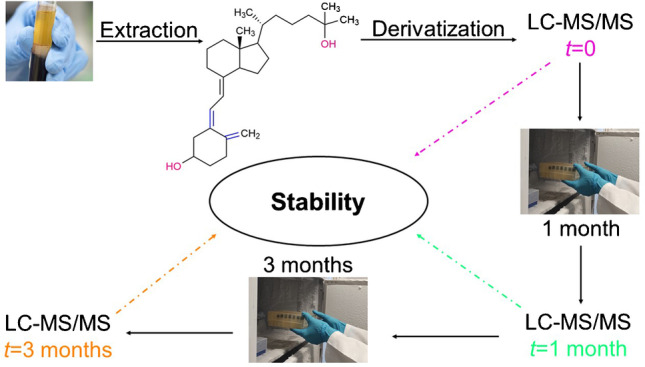

**Supplementary Information:**

The online version contains supplementary material available at 10.1007/s00216-022-04409-5.

## Introduction

Measurement of vitamin D metabolites requires selective and sensitive assays that enable accurate measurement of very low concentrations of multiple species in biological sample matrices, which presently can only be achieved by liquid chromatography/tandem mass spectrometry (LC–MS/MS) [1]. Usually, vitamin D measurements are performed in blood-based matrices such as serum or plasma, but there is a variety of other biological samples (urine, saliva, tissues, etc.), where vitamin D metabolites are investigated [2]. A wide range of different sample preparation techniques are employed prior to instrumental analysis [3] and sometimes a chemical derivatization step is incorporated because it can enhance the assay’s sensitivity and selectivity, which is important for quantification of low abundant metabolites. Furthermore, chemical derivatization can also improve the chromatographic separation. Obviously, these chemical derivatization reactions are ideally fast and inexpensive and preferably result in only one stable product with high yields.

The stability of the analytes in the sample is an important factor, which is investigated during the assay validation. Guidelines for the investigation of stability are available from various regulatory bodies such as the Food and Drug Administration (FDA), US Pharmacopeia (USP), US Environmental Protection Agency (USEPA), American Association of Official Analytical Chemists (AOAC), European Medicines Agency (EMA) and Eurachem [4, 5]. Stability ensures that the concentration of the investigated compounds is not affected during sample preparation and sample analysis as well as the storage conditions of the sample or the extracted sample. There are subtle differences between these guidelines, but all of them aim to guarantee the quality of the analytical method [6–8]. FDA recommends the investigation of (1) autosampler stability, (2) bench-top stability, (3) extract (or processed sample) stability, (4) freeze–thaw stability, (5) long term stability and (6) stock solution stability [9]. According to EMA, for the stability of the sample, the following stability tests should be evaluated [10]: (1) stability of the stock solutions and working solutions of the analytes and internal standard, (2) freeze and thaw stability, (3) short-term stability of the analyte in matrix and (4) long-term stability of the analytes in matrix stored in the freezer. Additionally, for the stability of the extracted sample, (1) autosampler stability of the processed sample and (2) stability of the processed sample under the storage conditions to be used during the study until the sample will be analyzed should be evaluated.

If sample preparation requires a derivatization step, it must be ensured that the resulting derivatization products of the analytes are stable until the sample is measured. Usually, this stability test refers to the sample processing time, sample handling and waiting time in the autosampler. However, there are cases, e.g. instrument failures or need for intermediate storage, advance preparation of large sample numbers, prevention of the need to repeat derivatization for re-measurements etc., when the extracted sample must be stored in the freezer before measurement. As a consequence, it is essential that the derivatized analytes are sufficiently stable until the sample is measured. While a stable isotope standard for each analyte in the sample should ensure accuracy if analyte and stable isotope standard degrade to the same extent, significant degradation rates might lower the concentrations of low abundant metabolites to levels outside the validated calibration range or even below LOQ or LOD.

The stability of vitamin D_3_ metabolites in biological samples has been thoroughly investigated, mostly stabilities in sample matrices prior to sample preparation, in particular freeze–thaw stability [11–15], bench-top stability [15], long-term stability [15, 16] and stability regarding differently preserved blood samples (lithium heparin plasma, EDTA plasma) [13] and exposure to light and temperature [14]. Some studies also describe the stability of vitamin D_3_ metabolite derivatization products. Hedman et al. performed Amplifex derivatization of 1,25-dihydroxyvitamin D_3_ (1,25(OH)_2_D_3_) and 1,25-dihydroxyvitamin D_2_ (1,25(OH)_2_D_2_) and stabilized the products from thermal and photochemical degradation, making them stable for several months [17]. Bonnet et al. investigated the stability of Amplifex products in the autosampler (5 °C, 24 h) [18]. After 24 h, the signal decreased by − 15.3, − 19.7 and − 42.6%, for vitamin D_3_, 25-hydroxyvitamin D_3_ (25(OH)D_3_) and 1,25(OH)_2_D_3_, respectively. Fabregat-Cabello et al. re-injected extracted samples, which were derivatized with Amplifex, after leaving them in the autosampler for 24 h at 5 °C. The authors noticed no significant differences in peak areas [19]. For 4-phenyl-1,2,4-triazoline-3,5-dione (PTAD) derivatives, Lyu et al. observed no significant analyte loss when the extract was left for 24 h at room temperature [15]. Similarly, He et al. described stable extracts when left in the autosampler for 96 h at 4 °C [11].

To our knowledge, no systematic stability studies of chemical derivatization products of vitamin D_3_ metabolites have so far been conducted. In the present study, we investigated the stabilities of five different vitamin D_3_ metabolites in sample extracts after chemical derivatization, after 1 month of storage at − 20 °C. While processed sample extracts are unlikely to be stored for longer than one month, we also investigated the longer-term stability over 3 months, as comparative longer-term data are available for the stability of vitamin D metabolites in serum samples, even for years-long storage periods [20, 21]. Four dienophile reagents, namely (i) PTAD; (ii) 4-[2-(6,7-dimethoxy-4-methyl-3-oxo-3,4-dihydroquinoxalyl)ethyl]-1,2,4-triazoline-3,5-dione (DMEQ-TAD); (iii) Amplifex; (iv) 2-nitrosopyridine, two chemical reagents for hydroxyl groups; (v) isonicotinoyl chloride (INC); (vi) 2-fluoro-1-methylpyridinium p-toluenesulfonate (FMP-TS); and (vii) a combination of reagents, which included PTAD derivatization and acetylation of the hydroxyl groups using acetic anhydride, were systematically investigated in this work.

## Material and methods

### Chemicals and reagents

The investigated reagents for the chemical derivatization reactions were PTAD, FMP-TS (< 5% 2-hydroxy-1-methylpyridinium p-toluenesulfonate, technical grade, ≥ 90%), isonicotinoyl chloride hydrochloride (95%), acetic anhydride (≥ 99%), pyridine (anhydrous, 99.8%), acetonitrile (ACN) (anhydrous, 99.8%); 4-dimethylaminopyridine (DMAP) (99%) obtained from Sigma-Aldrich (Steinheim, Germany); acetic acid and triethylamine (≥ 99,5%) from Carl Roth GmbH & Co. KG (Karlsruhe, Germany); DMEQ-TAD from Enzo Life Sciences (City, NY, USA); 2-nitrosopyridine from MedChemExpress (City, NJ, USA); and Amplifex Diene Reagent Kit from Sciex (Darmstadt, Germany).

Standards were purchased as follows: 3β-25(OH)D_3_ and 1,25(OH)_2_D_3_ from Cayman Chemical (Ann Arbor, MI, USA); vitamin D_3_ and 24,25-dihydroxyvitamin D_3_ ((24R)-24,25(OH)_2_D_3_) from Toronto Research Chemicals (Toronto, ON, Canada); and 3α-25(OH)D_3_ from Sigma-Aldrich (Steinheim, Germany).

Acetonitrile and methanol (MeOH) (UHPLC-MS grade) were obtained from Chemsolute (Th. Geyer GmbH & Co. KG, Renningen, Germany), while formic acid (97%) was from Alfa Aesar (Karlsruhe, Germany). A Millipore (Bedford, MA, USA) Direct-Q8 purification system was used to produce organic free water.

The human vitamin D_3_ free serum was purchased from Sigma-Aldrich (VD-DDC Mass Spect Gold serum).

### Preparation of standard solutions and sample preparation of serum samples

Stock solutions of the investigated vitamin D_3_ compounds were prepared in methanol at 1 mg/mL and stored at − 20 °C. These solutions were used to spike serum samples with the investigated compounds. Human vitamin D_3_ free serum was used and divided into two groups after spiking: group A included 3β-25(OH)D_3_ at 20 ng/mL, vitamin D_3_ at 30 ng/mL and 1,25(OH)_2_D_3_ at 10 ng/mL; group B included 3α-25(OH)D_3_ at 5 ng/mL and 24,25(OH)_2_D_3_ at 10 ng/mL. The reason for the separation into two groups was the lack of adequate chromatographic separation of 25(OH)D_3_ epimers and dihydroxy species. The concentrations of the metabolites were chosen sufficiently high to be able to be measure even without derivatization.

The sample preparation protocol was previously described by Ding et al. [22]. Briefly, protein precipitation was performed in 100 μL of serum using 250 μL of acetonitrile followed by 1 min vortexing and 15 min of centrifugation. A Concentrator plus/Vacufuge® plus (Eppendorf, Hamburg, Germany) was used to evaporate the supernatant to dryness after its transfer to a new vial and two-step liquid–liquid extraction (LLE): firstly, 100 μL of water and 200 μL ethyl acetate were added to the dry residue followed by 30 s vortexing and 5 min centrifugation. After removing the upper organic phase to a fresh vial, the aqueous phase was re-extracted by adding 200 μL of ethyl acetate. From the two combined organic phases, 380 μL were transferred to a new vial and evaporated to dryness. The dry residue was used for chemical derivatization, the details of which are described in the following section.

For every derivatization reagent that was examined, three spiked serum samples from group A and three spiked serum samples from group B were derivatized. Every sample was measured in triplicate. Moreover, a blank sample that underwent the identical sample preparation and derivatization steps was prepared for all reagents and was measured in triplicate.

The coefficient of variation (RSD) in the stability testing was between ± 5%, ± 8% and ± 7% for all metabolite derivatization products at *t* = 0, after 1 month and after 3 months, respectively. The calculated RSD values exhibited very good reproducibilities, ensuring that loss of observed analyte signals could be confidently assigned to sample degradation.

### Derivatization procedures

The individual steps of the derivatization reactions are compared in Table 1 for the single incubation reactions and in Table 2 for the double incubation reactions. The FMP-TS, INC and PTAD-Ac derivatization reactions were developed by our group to further enhance the detection sensitivity.

**Table 1:** Description of single incubation chemical derivatization reactions

	PTAD **[**22**]**	Amplifex	FMP**-**TS	INC	PyrNO [23]
1. Reagent preparation	0.5 mg/mL PTAD in dry ACN	Prepare Amplifex reagent as suggested by manufacturer	5 mg/mL FMP-TS in dry ACN + 1% triethylamine	5 mg/mL INC in ACN 10 mg/mL DMAP in ACN	0.27 mg/mL PyrNO in MeOH
2. Reagent addition to the dry sample residue	50 μL PTAD	50 μL Amplifex	50 μL FMP-TS + 1% triethylamine	100 μL ACN 10 μL INC 10 μL DMAP	40 μL PyrNO
3. Incubation conditions	RT, 60 min in darkness	RT, 60 min	40 °C, 15 min	10 s incubation while vortexing in RT	70 °C, 60 min
4. Reaction quenching	50 μL MeOH	50 μL MeOH	50 μL MeOH	-	-
5. Evaporation to dryness					
6. Sample reconstitution 100 μL of a mixture of MeOH:H_2_O (9:1, v/v)

**Table 2:** Description of double incubation chemical derivatization reactions

	PTAD **+ **acetylation	DMEQ**-**TAD [24]
1. Reagent preparation	A1: 0.5 mg/mL PTAD in dry ACN + 2% acetic acid A2: pyridine:acetic anhydride (2:1,v/v) including 2 mg/mL of DMAP	0.1 mg/mL DMEQ-TAD in ethyl acetate
2. 1st Reagent addition to the dry sample residue	33.3 μL of A1	25 μL DMEQ-TAD
3. 1st Incubation conditions	RT, 60 min in darkness	RT, 30 min
4. 2nd Reagent addition	33.3 μL of A2	25 μL DMEQ-TAD
5. 2nd Incubation conditions	RT, 60 min in darkness	RT, 60 min
6. Reaction quenching	50 μL MeOH	50 μL MeOH
7. Evaporation to dryness		
8. Sample reconstitution 100 μL of a mixture of MeOH:H_2_O (9:1, v/v)		

### Design of the stability experiments

To evaluate the storage stability of the vitamin D_3_ metabolites’ derivatization products, three spiked serum samples were prepared independently for the chosen analytes’ concentration level (see above). Every sample was measured in triplicate. Moreover, we also examined the stability of the non-derivatized metabolites. We limited the extent of the study to one concentration level, as otherwise it would have been too time-consuming. We chose the concentration level to be as close as possible to typical concentrations in serum samples, but at the same time still quantifiable as underivatized compounds. The long-term stability testing was carried out by storing the samples at − 20 °C for 1 and 3 months. The samples corresponding to each derivatization reagent were measured at three time points; (1) freshly prepared, *t* = 0; (2) after 1 month of storage at − 20 °C; and (3) after 3 months of storage at − 20 °C.

### Liquid chromatography-tandem mass spectrometry

For, LC–MS/MS, 5 μL of each sample solution were injected into a 1290 Infinity II LC system (Agilent, CA, USA). A Phenomenex (Torrance, CA, USA) Kinetex 2.6 µm C-18 100 Å column (100 × 2.1 mm). The mobile phases consisted of solvent (A) water (+ 0.1% formic acid) and solvent (B) methanol (+ 0.1% formic acid). A gradient elution program was applied for 15 min and solvent (B) increased linearly from 50 to 100%. Subsequently, it was held constant for 2 min at 100% (B) before returning to the initial conditions. Re-equilibration was performed for 3 min. The flow rate of the mobile phase was constant at 0.4 mL/min, and the temperature in the column oven was set at 30 °C. The same elution program was used for all the tested reagents. The slow gradient was chosen to allow optimum separation of the epimers, isomers and stereoisomeric derivatization products. Importantly, this HPLC method was only used for the purpose of the stability study. It is not a routine method optimized for routine, high-throughput analyses of vitamin D metabolites.

The UHPLC system was coupled to a Sciex (Concord, ON, Canada) QTRAP 6500 + triple quadrupole-linear ion trap mass spectrometer equipped with a Turbo-V electrospray ion source in positive ion mode. Data acquisition was performed in MRM mode. The parameters of the ion source were as follows: curtain gas, 35 psi; IonSpray voltage, 5500 V; nebulizer gas, 30 psi; heating gas, 30 psi; collision gas was set to medium. MRM transitions and collision-induced dissociation conditions were optimized for each vitamin D metabolite derivatization product individually. The optimized values for declustering potential (DP), entrance potential (EP), collision energy (CE) and collision cell exit potential (CXP) are summarized in Table S1 (Supplementary Material). The dwell time was chosen to provide 12–20 data points across the chromatographic peaks. Analyst (Sciex) version 1.7 and MultiQuant (Sciex) version 3.0.3 software were used for data analysis.

## Results and discussion

In the following section, we present the results of the long-term stability experiments of the different chemical derivatization products of vitamin D metabolites. Three time points were tested (*t* = 0, after 1 and 3 months) while the derivatized sample extracts were kept at − 20 °C. We chose this storage temperature because − 20 °C freezers are more commonly available in analytical laboratories than − 80 °C freezers. The mean measured signal intensities were compared to the mean signal intensities of the freshly prepared samples at *t* = 0. (Table 3).

**Table 3:** Long-term stability of five non-derivatized vitamin D_3_ metabolites and their corresponding derivatization products in spiked serum samples extracts (the results are expressed relative to the values at *t* = 0)

Metabolite	*t* = 0	After 1 month	After 3 months	Metabolite	*t* = 0	After 1 month	After 3 months
1,25(OH)_2_D_3_	100	70	34	1,25(OH)_2_D_3_-INC	100	80	83
24,25(OH)_2_D_3_	100	48	36	24,25(OH)_2_D_3_-INC	100	71	63
3β-25(OH)D_3_	100	58	48	3β-25(OH)D_3_-INC	100	62	61
3α-25(OH)D_3_	100	57	37	3α-25(OH)D_3_-INC	100	62	62
D_3_	100	90	68	D_3_-INC	100	100	37
1,25(OH)_2_D_3_-Amplifex	100	86	83	1,25(OH)_2_D_3_-PTAD	100	31	16
24,25(OH)_2_D_3_-Amplifex	100	89	86	24,25(OH)_2_D_3_-PTAD	100	37	24
3β-25(OH)D_3_-Amplifex	100	84	73	3β-25(OH)D_3_-PTAD	100	28	15
3α-25(OH)D_3_-Amplifex	100	82	73	3α-25(OH)D_3_-PTAD	100	28	21
D_3_-Amplifex	100	80	65	D_3_-PTAD	100	46	25
1,25(OH)_2_D_3_-DMEQ-TAD	100	79	66	1,25(OH)_2_D_3_-PTAD-Ac	100	82	56
24,25(OH)_2_D_3_-DMEQ-TAD	100	98	80	24,25(OH)_2_D_3_-PTAD-Ac	100	76	57
3β-25(OH)D_3_-DMEQ-TAD	100	77	61	3β-25(OH)D_3_-PTAD-Ac	100	81	57
3α-25(OH)D_3_-DMEQ-TAD	100	88	77	3α-25(OH)D_3_-PTAD-Ac	100	75	56
D_3_-DMEQ-TAD	100	75	50	D_3_-PTAD-Ac	100	75	65
1,25(OH)_2_D_3_-FMP	100	76	65	1,25(OH)_2_D_3_-PyrNO	100	68	59
24,25(OH)_2_D_3_-FMP	100	85	63	24,25(OH)_2_D_3_-PyrNO	100	65	59
3β-25(OH)D_3_-FMP	100	76	60	3β-25(OH)D_3_-PyrNO	100	61	57
3α-25(OH)D_3_-FMP	100	77	63	3α-25(OH)D_3_-PyrNO	100	61	55
D_3_-FMP	100	90	66	D_3_-PyrNO	100	0	0

Initially, we investigated the stability of the non-derivatized metabolites in serum after sample preparation. The extracted non-derivatized metabolites were not particularly stable, even after 1 month of storage at − 20 °C. The extracts exhibited 48–90% of the values seen for *t* = 0 (Table 3), and only vitamin D_3_ was sufficiently stable after 1 month of storage maintaining 90% of the observed value at *t* = 0.

Very poor stabilities after only 1 month of storage were seen for products of PTAD (28–46%) and PyrNO (0–68%). Aronov et al. observed no significant loss of PTAD products of 1,25(OH)_2_D_3_ and 25(OH)D_3_ standard solutions after one week at − 80, − 20, + 4 °C and at room temperature [25].

Wan et al. mentioned that the 1,25(OH)_2_D_3_-PyrNO derivatization product was stable for 1 week when kept at − 20 and − 80 °C with values observed ˃90% of those at *t* = 0 [23]. Importantly, these observations were for standard solutions, not for serum extracts as investigated in this study. Our extracted 1,25(OH)_2_D_3_-PyrNO derivatization product was only 68% of the signal seen for the freshly prepared sample after 1 month of storage at − 20 °C, which may be due to the longer storage time as well as interactions with co-extracted sample matrix compounds. In addition, Helmeczi et al. investigated the stability of the extracted 25(OH)D-PyrNO derivatization product after 2 freeze–thaw cycles when stored at − 80 °C [26]. The derivatization product was stable and no degradation was observed. The exact storage time of the extracts at − 80 °C during the two freeze–thaw cycles was not mentioned in the study [26].

The stabilities of DMEQ-TAD products were between 75 and 98%, FMP-TS were 76–90%, INC were 62–100%, and PTAD-Ac were 75–82% after 1 month of storage. DMEQ-TAD products were the most stable after 3 months of storage (50–80%). Faqehi et al. investigated short-term storage stability of estrogen-FMP derivatives at − 20 °C and − 80 °C after 24 h, 48 h and 28 days [27]. During the first 24 h, no significant reduction of the original response was observed. Degradation was described as significant after 48 h at − 20 °C (58–72%).) Finally, at − 80 °C, the stability was acceptable (91–94%) even after 28 days.

Noteworthy is the difference between the observed stabilities of products of PTAD and PTAD-Ac. The results clearly show that the additional acetylation improves the stabilities from 28–46% to 75–82% after 1 month of storage and from 15–25 to 56–65% after 3 months of storage.

In summary, the most stable extracted metabolite products were formed by Amplifex, which after 1 month of storage were within 80–89% of the *t* = 0 value and after 3 months were within 65–86% of *t* = 0. The 24,25(OH)_2_D_3_-Amplifex products were the most stable in our study.

Adding a stable isotope internal standard for each metabolite would likely compensate for accuracy issues originating from analyte degradation, assuming that the degradation rates are the same for all species, which is a reasonable assumption. However, vitamin D metabolites present in the sample at very low concentration levels, e.g. those seen for 1,25(OH)_2_D_3_ or 3α-25(OH)D_3_, which undergo very high degradation (˃30%) at the same time (e.g. PTAD or PyrNO derivatization products), can potentially diminish to very low concentration levels in the stored extracts, which are then below the validated calibration range or even below LOQ or LOD. The assay then is not fit for purpose anymore.

The EMA and FDA guidelines prescribe that analyte stabilities in the samples should be within ± 15% of the values at *t* = 0 after the chosen storage conditions and time period [9, 10]. Considering this criterion in our comparison study, it allows us to identify the optimum derivatization reagent for each vitamin D_3_ metabolite, if they were to be measured individually (Fig. S1 A–E  in the Supplementary Material provide a complete breakdown of the data). For 1,25(OH)_2_D_3_, Amplifex and INC the most stable products were provided after 3 months of storage, with PTAD-Ac products being equally stable, but only for 1 month of storage. Amplifex is the best choice for 24,25(OH)_2_D_3_, since even after 3 months of storage the extract met the 15% criterion. Similarly, the stability cut-off is also met for storage of 24,25(OH)_2_D_3_ FMP-TS and DMEQ-TAD derivatization products after 1 month. Amplifex met the criterion for 3β-25(OH)D_3_ and DMEQ-TAD for 3α-25(OH)D_3_ after 1 month. Vitamin D_3_ exhibited the largest variation of stabilities. INC derivatives were the most stable after 1 month (100%), while after 3 months, the stability dropped to only 37% of the *t* = 0 value. Moreover, Vitamin D_3_-PyrNO completely degraded to 0% already after 1 month of storage. Finally, non-derivatized vitamin D_3_ as well as the FMP-TS product of vitamin D_3_ degraded less than 15% after 1 month of storage.

## Conclusions

Stability studies should investigate the preferred storage conditions over storage periods that are equal or exceed those applied to real samples. Usually, the only criterion for the stability that guidelines specify is that the value after a certain period of time or under certain conditions of storage should be within ± 15% of the value at the start (*t* = 0) [9, 10]. However, a laboratory can set more strict criteria, such as ± 10% or even ± 5% to ensure the validity of their analytical method [7].

Taking the above into consideration, the most stable chemical derivatives of the investigated vitamin D_3_ metabolites were formed by the Amplifex reagent, which exhibited good stability within the ± 15% range during the first 1 month of storage; for some analytes even after 3 months. Similarly good stability was observed for some vitamin D metabolite derivatization products of DMEQ-TAD, FMP-TS, INC and PTAD-Ac after 1 month (± 15%). Very poor stability, even after the first month, was seen for the PTAD and PyrNO derivatization products. None of them complied with the given stability criterion.

In comparison to the stability of native, protein-bound vitamin D metabolites in blood serum or plasma, however, these values are somewhat poor, showing that derivatized metabolites are generally much less stable and should not be stored for extended periods of time at − 20 °C. Storing fully derivatized sample extracts at − 20 °C should only be used as an emergency measure in case of instrument failure or if extracts are to be used within a short period of time, e.g. within 1 week. Hollis reported no detectable degradation for 25(OH)D and 1,25(OH)_2_D in pooled human samples stored for over 10 years at − 20 °C [28]. Serum samples stored for 6, 24 or 40 years are reported to have been re-analysed with no significant effect on 25(OH)D levels [20, 21]. Even under extreme storage conditions, serum 25(OH)D_2_ and 25(OH)D_3_ showed very small to no degradation unless they were subjected to prolonged exposure to direct sunlight [29].

In summary, extracts from serum samples containing vitamin D_3_ metabolites can be remeasured after 1 or even 3 months storage at − 20 °C, if the proper derivatization reagent is chosen and a small degree of degradation is accepted. An appropriate stable isotope internal standard will then compensate for the degradation in most cases. Storage of derivatized sample extracts is important as it allows remeasurement of selected samples or entire batches if, for example, the instrument breaks down or samples are shipped to another laboratory. It also permits automated, parallel sample processing, including the derivatization step, of large sample numbers, which requires safe storage of samples prior to instrumental analysis.

## Supplementary Information

Below is the link to the electronic supplementary material.

Supplementary file1 (DOCX 50 KB)
