# Supplementary material for: Stability of sample extracts of vitamin D3 metabolites after chemical derivatization for LC–MS/MS analysis
Source: Anal Bioanal Chem. 2022 Nov 7;415(2):327–33. doi: 10.1007/s00216-022-04409-5 (PMC9823060; doi:10.1007/s00216-022-04409-5)
Supplement: Supplementary file 1 — Supplementary file1 (DOCX 50 KB) [file 216_2022_4409_MOESM1_ESM.docx]

*Supplementary material*

**Stability of sample extracts of vitamin D_3_ metabolites after chemical derivatization for LC-MS/MS analysis**

**Anastasia Alexandridou^1^, Dietrich A. Volmer^1^***

**^1^***Bioanalytical Chemistry, Department of Chemistry, Humboldt University Berlin, Brook-Taylor-Str. 2, 12489 Berlin, Germany*

*Corresponding author:

Prof. Dr. Dietrich Volmer

Humboldt University Berlin

Department of Chemistry, 12489 Berlin, Germany

Tel +49 30 2093 7588

Email: [Dietrich.Volmer@hu-berlin.de](mailto:Dietrich.Volmer@hu-berlin.de)

**Table S1.** Optimized MS/MS (MRM) settings for the vitamin D_3_ metabolites and their derivatization products.

| **Compound** | **Q1 (*m/z*)** | **Q3 (*m/z*)** | **DP (V)** | **EP (V)** | **CE (V)** | **CXP (V)** |
| --- | --- | --- | --- | --- | --- | --- |
| IS: 25(OH)D_3_-d6 | 407.4 | 389.3 | 40 | 11 | 12 | 15 |
|  | 407.4 | 371.4 | 40 | 11 | 14 | 15 |
| 3β-/3α-25(OH)D_3_ | 401.4 | 383.3 | 40 | 11 | 12 | 15 |
|  | 401.4 | 365.4 | 40 | 11 | 14 | 15 |
|  | 401.4 | 159.1 | 40 | 11 | 30 | 17 |
| 24,25(OH)_2_D_3_ | 417.4 | 399.3 | 50 | 11 | 12 | 14 |
|  | 417.4 | 381.3 | 50 | 11 | 12 | 12 |
|  | 417.4 | 363.3 | 50 | 11 | 15 | 12 |
| 1,25(OH)_2_D_3_ | 417.4 | 399.3 | 150 | 11 | 9 | 13 |
|  | 417.4 | 381.3 | 150 | 11 | 13 | 12 |
|  | 417.4 | 363.3 | 150 | 11 | 14 | 14 |
| D_3_ | 385.5 | 367.3 | 115 | 13 | 15 | 15 |
|  | 385.5 | 259.5 | 115 | 13 | 16 | 9 |
|  | 385.5 | 159.0 | 115 | 13 | 31 | 19 |
| 3β-/3α-25(OH)D_3_-PTAD | 558.4 | 298.3 | 155 | 8 | 21 | 9 |
|  | 558.4 | 280.3 | 155 | 8 | 38 | 10 |
|  | 558.4 | 161.1 | 155 | 8 | 50 | 19 |
| 24,25(OH)_2_D_3_-PTAD | 592.3 | 298.2 | 70 | 12 | 24 | 9 |
|  | 592.3 | 280.1 | 70 | 12 | 40 | 13 |
|  | 592.3 | 161.0 | 70 | 12 | 52 | 18 |
| 1,25(OH)_2_D_3_-PTAD | 592.3 | 314.2 | 45 | 12 | 23 | 14 |
|  | 592.3 | 268.2 | 45 | 12 | 44 | 12 |
|  | 592.3 | 177.2 | 45 | 12 | 46 | 9 |
| D_3_-PTAD | 560.4 | 365.3 | 85 | 14 | 26 | 12 |
|  | 560.4 | 298.2 | 85 | 14 | 26 | 13 |
|  | 560.4 | 280.2 | 85 | 14 | 50 | 18 |
| 3β-/3α-25(OH)D_3_-PTAD+Ac | 600.3 | 340.3 | 125 | 12 | 18 | 10 |
|  | 600.3 | 280.1 | 125 | 12 | 32 | 13 |
|  | 600.3 | 161.1 | 125 | 12 | 50 | 18 |
| 24,25(OH)_2_D_3_-PTAD+Ac | 676.3 | 340.2 | 45 | 11 | 24 | 10 |
|  | 676.3 | 280.1 | 45 | 11 | 37 | 13 |
|  | 676.3 | 161.1 | 45 | 11 | 59 | 19 |
| 1,25(OH)_2_D_3_-PTAD+Ac | 676.3 | 598.3 | 45 | 10 | 16 | 23 |
|  | 676.3 | 658.3 | 45 | 10 | 9 | 24 |
|  | 676.3 | 398.3 | 45 | 10 | 21 | 14 |
| D_3_-PTAD+Ac | 602.3 | 542.6 | 200 | 12 | 21 | 18 |
|  | 602.3 | 340.2 | 200 | 12 | 19 | 14 |
|  | 602.3 | 280.2 | 200 | 12 | 35 | 13 |
| 3β-/3α-25(OH)D_3_-Amplifex | 732.5 | 673.3 | 110 | 11 | 43 | 22 |
|  | 732.5 | 275.2 | 110 | 11 | 66 | 14 |
|  | 732.5 | 217.2 | 110 | 11 | 69 | 20 |
| 24,25(OH)_2_D_3_-Amplifex / 1,25(OH)_2_D_3_-Amplifex | 748.6 | 689.5 | 120 | 11 | 40 | 22 |
|  | 748.6 | 275.2 | 120 | 11 | 70 | 13 |
|  | 748.6 | 217.2 | 120 | 11 | 70 | 10 |
| D_3_-Amplifex | 716.5 | 657.5 | 85 | 12 | 43 | 21 |
|  | 716.5 | 275.1 | 85 | 12 | 68 | 13 |
|  | 716.5 | 217.3 | 85 | 12 | 68 | 19 |
| 3β-/3α-25(OH)D_3_-PyrNO | 509.3 | 491.3 | 120 | 11 | 28 | 16 |
|  | 509.3 | 473.3 | 120 | 11 | 30 | 16 |
|  | 509.3 | 231.2 | 120 | 11 | 29 | 11 |
| 24,25(OH)_2_D_3_-PyrNO / 1,25(OH)_2_D_3_-PyrNO | 525.4 | 507.4 | 120 | 11 | 27 | 16 |
|  | 525.4 | 489.3 | 120 | 11 | 30 | 19 |
| 24,25(OH)_2_D_3_-PyrNO | 525.4 | 231.2 | 120 | 11 | 30 | 11 |
| 1,25(OH)_2_D_3_-PyrNO | 525.4 | 247.2 | 120 | 12 | 29 | 11 |
| D_3_-PyrNO | 493.6 | 475.3 | 130 | 12 | 26 | 15 |
|  | 493.6 | 231.2 | 130 | 12 | 28 | 10 |
| 3β-/3α-25(OH)D_3_-DMEQ-TAD | 746.5 | 468.3 | 80 | 10.5 | 38 | 15 |
|  | 746.5 | 247.2 | 80 | 10.5 | 53 | 11.5 |
|  | 746.5 | 203.1 | 80 | 10.5 | 120 | 24 |
| 24,25(OH)_2_D_3_-DMEQ-TAD / 1,25(OH)_2_D_3_-DMEQ-TAD | 762.5 | 468.3 | 120 | 10.5 | 38 | 15 |
|  | 762.5 | 247.2 | 120 | 10.5 | 54 | 11.5 |
|  | 762.5 | 203.1 | 120 | 10.5 | 125 | 23 |
| D_3_-DMEQ-TAD | 730.5 | 468.3 | 150 | 10.5 | 34 | 15 |
|  | 730.5 | 247.2 | 150 | 10.5 | 50 | 28 |
|  | 730.5 | 203.1 | 150 | 10.5 | 115 | 23.5 |
| 3β-/3α-25(OH)D_3_-FMP | 492.3 | 383.2 | 35 | 13 | 12 | 13 |
|  | 492.3 | 365.2 | 35 | 13 | 18 | 13 |
|  | 492.3 | 159.1 | 35 | 13 | 35 | 17 |
| 24,25(OH)_2_D_3_- FMP / 1,25(OH)_2_D_3_- FMP | 508.3 | 399.3 | 35 | 13 | 13 | 15 |
|  | 508.3 | 381.3 | 35 | 13 | 18 | 16 |
|  | 508.3 | 363.3 | 35 | 13 | 20 | 13 |
| D_3_-FMP | 476.3 | 367.3 | 30 | 13 | 14 | 12 |
|  | 476.3 | 159.1 | 30 | 13 | 37 | 18 |
|  | 476.3 | 145.0 | 30 | 13 | 40 | 17 |
| 3β-/3α-25(OH)D_3_-INC | 506.4 | 488.3 | 110 | 10 | 21 | 16 |
|  | 506.4 | 365.3 | 110 | 10 | 25 | 16 |
|  | 506.4 | 124.0 | 110 | 10 | 32 | 15 |
| 24,25(OH)_2_D_3_-INC / 1,25(OH)_2_D_3_-INC | 522.4 | 504.4 | 95 | 11 | 23 | 17 |
|  | 522.4 | 124 | 95 | 11 | 33 | 15 |
| 24,25(OH)_2_D_3_-INC | 522.4 | 486.4 | 110 | 12 | 27 | 15 |
| 1,25(OH)_2_D_3_-INC | 522.4 | 381.3 | 85 | 10 | 25 | 12 |
| D_3_-INC | 490.3 | 124.0 | 200 | 10 | 28 | 14 |
|  | 490.3 | 367.3 | 200 | 10 | 21 | 16 |

**Figure S1 A-E.** Comparison of the long-term stability of each vitamin D_3_ metabolite and their derivatization products in plasma sample extracts.
